# High expression of protein phosphatase 4 is associated with the aggressive malignant behavior of colorectal carcinoma

**DOI:** 10.1186/s12943-015-0356-7

**Published:** 2015-04-28

**Authors:** Xinxiang Li, Lei Liang, Liyong Huang, Xiaoji Ma, Dawei Li, Sanjun Cai

**Affiliations:** Department of Colorectal Surgery, Fudan University Shanghai Cancer Center, Shanghai, 200032 China; Department of Oncology, Shanghai Medical College, Fudan University, Shanghai, 200032 China

**Keywords:** Colorectal carcinoma, PP4C, Cell invasion, Prognosis, AKT

## Abstract

**Background:**

Recent evidence suggests an important role of protein phosphatase 4 (PP4C) in the progression of several cancers, including breast cancer, lung cancer and pancreatic ductal adenocarcinoma. However, the contribution of PP4C to colorectal carcinoma (CRC) remains elusive.

**Methods:**

The expression of PP4C in CRC tissues compared with matched non-tumor tissues and CRC cells was detected using quantitative RT-PCR, immunohistochemistry and western blotting assays. Through univariate and Kaplan-Meier analysis, we correlated the PP4C expression with clinicopathological features and patient survival. A series of experiments, including cell proliferation, lentiviral infection, cell invasion and MMP gelatinase activity assays, were performed to investigate the underlying mechanisms. Through further experiments, tumor growth and metastasis were evaluated *in vivo* using a xenogenous subcutaneously implant model and a tail vein metastasis model.

**Results:**

In the present study, we found that PP4C expression is frequently increased in human CRC and that the upregulation of PP4C correlates with a more invasive tumor phenotype and poor prognosis. The ectopic expression of PP4C promoted CRC cell proliferation, migration and invasion *in vitro* and tumor growth and lung metastasis *in vivo*. Silencing the expression of PP4C resulted in the inhibition of cell proliferation and invasion. Further investigations showed that phosphorylated Akt (p-AKT) is required for the PP4C-mediated upregulation of MMP-2 and MMP-9, which promotes cell invasion.

**Conclusions:**

Our data suggested a potential role of PP4C in tumor progression and provided novel insights into the mechanism of how this factor positively regulated cell proliferation and invasion in CRC cells.

## Background

Colorectal cancer (CRC) is a common cancer and a leading cause of cancer-related mortality and morbidity worldwide. The incidence of CRC is increasing [1,2]. The current standard treatment for patients with CRC is surgical resection followed by adjuvant chemotherapy. However, the overall survival rate of patients varies from 90% to less than 5% due to different stages in the disease progress at diagnosis [3,4]. Post-operative recurrence and metastasis play a crucial role in the progression of CRC. However, the multiple alterations of molecular mechanisms that lead to abnormal changes in signaling pathways and malignant behaviors remain unclear [5,6]. Therefore, understanding the key factors in these processes is crucial for finding new therapeutic molecular targets of CRC.

Accumulating evidence has demonstrated that aberrant gene expression leads to high tumorigenic properties by affecting the control of growth, apoptosis, differentiation, migration and invasion [7-9]. Protein phosphatase 4 (PP4C), a member of the PPP family of serine/threonine protein phosphatases, contributes to the nucleation, growth and maturation of the centrosome during cell division [10-12]. Studies have demonstrated that PP4C played diverse cellular functions in different organ or cell lines. For example, the overexpression of PP4C reduces the colony-forming ability and induces apoptosis of mouse thymoma cells and HEK 293 T cells [13,14]. However, increasing evidence indicates the important role of PP4C in human tumorigenesis. Suppressing the expression of PP4C induces apoptosis in A549 and HeLa cells [15]. Increased expression of PP4C has been observed in breast cancer, lung cancer and pancreatic ductal adenocarcinoma [16,17]. In addition, the overexpression of PP4C regulates the NF-κB, JNK and m-TOR pathway [18-20]. These studies indicate that PP4C plays an important role in cancer development. Despite the increased overexpression of PP4C in human cancers, the molecular mechanism underlying the role of PP4C in CRC remains to be elucidated.

AKT has been reported to play important roles in a variety of cellular processes that are considered to be cancer hallmarks [21,22]. Several studies have reported that the AKT proto-oncogene is overexpressed and required for cell proliferation, angiogenesis, invasion and metastasis in CRC [23,24]. It is tempting to speculate that the upregulation of PP4C expression in CRC may be responsible for the increased AKT-mediated responses resulting in cell survival and invasion, a phenomenon that is known to occur in cancer.

In the present study, we analyzed the PP4C expression levels in CRC tissues compared with paired non-tumor tissues and evaluated the correlations between the PP4C level and clinicopathological features. The overexpression of PP4C promoted cancer cell growth and invasion and markedly increased the MMP-2/MMP-9 activities through a PI3K/AKT-dependent signaling pathway. This study provides the first demonstration that the overexpression of PP4C promotes tumorigenesis and lung metastasis *in vivo*.

## Results

### PP4C is upregulated in colorectal cancer tissues and cell lines

**Figure 1:**
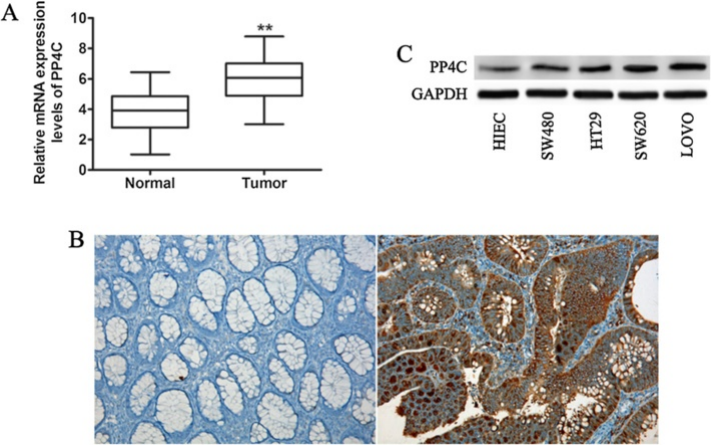
PP4C was overexpressed in CRC tissues and cell lines. **(A)** The PP4C mRNA expression in CRC specimens and paired adjacent non-cancer colorectal tissues was determined by qRT-qPCR. **(B)** Representative images show PP4C expression in normal tissues adjacent to colorectal tissue (left) and colorectal cancer (right) (Original magnification, 100×). **(C)** Western blotting analysis of PP4C expression in five CRC cell lines. ** represents p < 0.01.

Previous studies have indicated that the expression of PP4C is upregulated in tumors of different origins, including breast cancer, lung cancer and pancreatic ductal adenocarcinoma [16,17]. To explore the expression of PP4C in CRC, we assessed the mRNA levels of PP4C in a cohort of 92 CRC patients with paired adjacent non-tumorous tissue by qRT-PCR. The results revealed that the mRNA expression level of PP4C was significantly upregulated in tumor tissues compared with the level in matched non-tumor tissues (Figure 1A). To confirm this observed upregulation at protein level, we detected the PP4C expression levels using immunohistochemistry. As shown in Figure 1B, representative immunohistochemical staining results demonstrated that the PP4C protein expression in the tissue samples adjacent to CRC tumors was negative or low but significantly higher in the CRC tumors.

**Figure 2:**
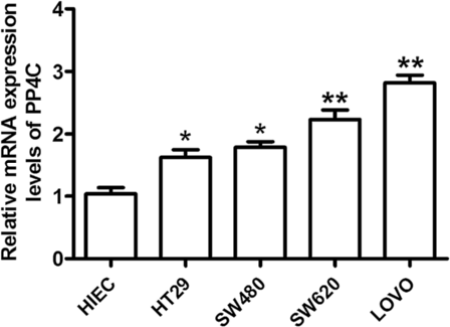
PP4C expression was determined in an immortalized human intestinal epithelial cell line (HIEC) and various CRC cell lines by real-time PCR analysis.

To further examine whether CRC cells present elevated PP4C expression levels, we compared the expression of PP4C among an immortalized intestinal epithelial cell line (HIEC) and CRC cell lines (SW480, HT29, SW620 and LOVO) at both the mRNA and protein levels. Interestingly, of the five cell lines tested, the protein expression level of PP4C was significantly higher in the more tumorigenic and invasive SW620 and LOVO cell lines. PP4C was lower in the less tumorigenic and noninvasive SW480 and HT29 cell lines and the normal intestinal epithelial cell line HIEC (Figure 1C). Consistently, qRT-PCR further confirmed the results from Western blotting analysis (Figure 2). Taken together, these data provide the initial evidence that PP4C is significantly upregulated in CRC tissues, indicating that PP4C may be associated with increased tumorigenicity and/or invasiveness, which play a crucial role in CRC.

### The expression level of PP4C is associated with clinicopathological features and prognosis in patients with colon cancer

**Figure 3:**
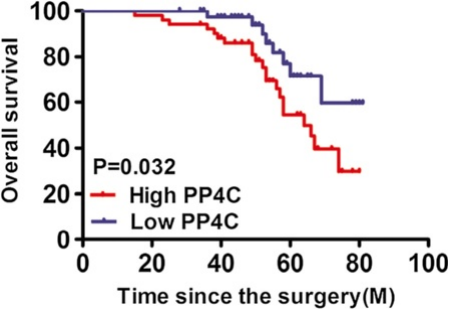
Increased PP4C expression was significantly associated with the overall survival (OS) of CRC patients. The data were analyzed using Kaplan-Meier survival analysis between patients with high PP4C expression and low PP4C expression according to the intensity scores.

The observed upregulated expression of PP4C in CRC prompted us to further investigate the clinical relevance of PP4C in the progression of CRC. The potential correlations of PP4C expression with a few clinicopathological parameters (Table 1) were examined in the patients. PP4C overexpression was associated with lymph node metastasis (P = 0.0013), vascular invasion (P = 0.0029), distant metastasis (P = 0.0081) and Dukes’ stage (P = 0.0048). However, no associations were found between PP4C expression and other clinical features, including age, gender, tumor location and tumor differentiation. Furthermore, Kaplan-Meier analysis using the log-rank test was performed, and the results showed that patients with high PP4C expression had a shorter median survival time of 60.4 months. Patients with low PP4C expression had a median survival time of 87.2 months (P = 0.032; Figure 3). Besides, multivariate analysis showed that PP4C expression served as an independent prognostic indicator for overall survival (OS) (Table 2).

**Table 1:** **Associations between PP4C expression levels and clinicopathologic features of colorectal cancer patient**

**Clinicopathologic Variables**		**PP4C expression**		**P**
	**N**	**Negative/Low**	**High**	
Gender				0.403
Female	59	34	25	
Male	33	17	16	
Age, y				0.251
≤50	34	20	14	
>50	58	31	27	
Tumor location				0.256
Colon	42	26	16	
Rectum	50	25	25	
Differentiation				0.413
Well	27	14	13	
Moderately	47	29	18	
Poorly	18	8	10	
Lymph node metastasis				0.0013*
Yes	27	8	19	
No	65	43	22	
Vascular invasion				0.0029*
Yes	28	9	19	
No	64	42	22	
Distant metastasis				0.0081*
Yes	11	2	9	
No	81	49	32	
AJCC stage				0.0108*
I-II	36	26	10	
III-IV	56	25	31	

Abbreviations: AJCC, American Joint Committee on Cancer (AJCC). *Statistically significant, P <0.05.

**Table 2:** **Multivariate analysis for OS**

**Variables**	**OS**	**P**
	**HR (95%CI)**	
Gender (Male vs Female)	0.56 (0.42-1.47)	0.264
Age (year, #0 vs >50)	0.58 (0.73-1.92)	0.227
Tumor location (colon vs rectum)	0.84 (0.86-1.46)	0.205
Lymph node metastasis (Yes vs No)	2.44 (1.69-5.42)	0.016*
Vascular invasion (Yes vs No)	2.27 (1.86-2.97)	0.035*
Distant metastasis (Yes vs No)	7.69 (3.27-16.38)	<0.001*
AJCC stage (I-II vs III-IV)	1.56 (1.21-4.85)	0.008*
PP4C expression (low vs high)	1.24 (1.02-3.59)	0.028*

Abbreviations: OS, overall survival; HR, hazard ratio; CI, Confidence interval. *Statistically significant, P <0.05.

### Ectopic expression of PP4C promotes cell growth and migration, whereas PP4C silencing inhibits CRC cell growth and invasion

**Figure 4:**
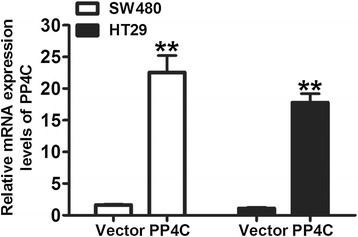
PP4C mRNA expression were measured through real-time PCR in SW480 and HT29 cells transfected with PP4C.

**Figure 5:**
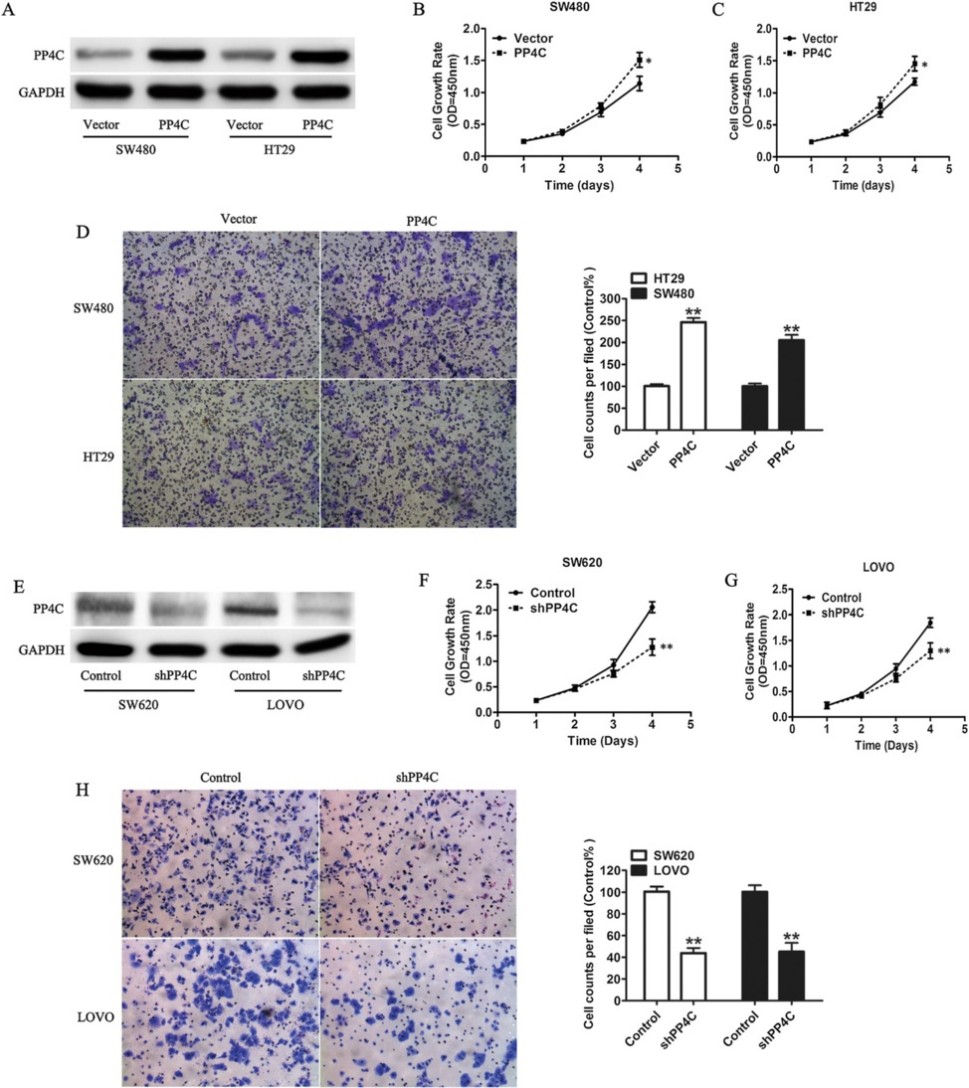
PP4C regulated CRC cell proliferation and invasion. **(A)** Western blotting analysis of PP4C in SW480 and HT29 cells transfected with PP4C or a vector control. **(B, C)** Effect of PP4C overexpression on cell proliferation was evaluated by CCK-8 assay [**(B)** SW480 and **(C)** HT29]. **(D)** Representative micrographs of invasion assay of the indicated cells at 24 h. **(E)** Western blotting analysis of PP4C in SW620 and LOVO cells transfected with shPP4C or a negative control. **(F, G)** Proliferation rates of cell sublines detected by CCK-8 assay after PP4C knockdown [**(F)** SW480 and **(G)** HT29]. **(H)** Representative micrographs of the indicated cells grown for 24 h on Matrigel in the invasion assay (Original magnification, 100× for **D** and **H**). The data are shown as the means ± SD of three independent experiments. *P < 0.05, **P < 0.01.

To determine whether PP4C possesses an oncogene function, PP4C was stably overexpressed through a lentiviral vector system in the SW480 and HT29 cell lines with low endogenous PP4C levels. We confirmed the PP4C expression levels through qRT-PCR (Figure 4) and Western blotting analysis (Figure 5A). To investigate whether PP4C plays a direct functional role in facilitating tumor cell growth and invasion in CRC cells, the cell proliferation was determined by CCK-8. The results showed that the overexpression of PP4C significantly promoted cell proliferation compared with the control cells (transfected with the vector control; Figure 5B and C). An invasion assay was performed to examine the invasive capability of cancer cells with upregulated PP4C. Increased invasion was observed in the PP4C-overexpressing cells compared with the vector control group (Figure 5D).

**Figure 6:**
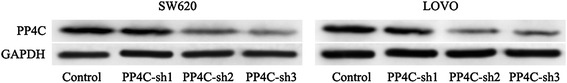
Knockdown efficiency was shown after treatment of PP4C shRNA in SW620 and LOVO cell lines, which presented high endogenous PP4C expression.

To further explore whether these aggressive cellular phenotypes were affected by PP4C in CRC cells, PP4C was knocked down by using a specific shRNA. Effective PP4C silencing was chosen for further experiments because it presented a better knockdown efficiency in both SW620 and LOVO cells and was denoted shPP4C (Figure 5E) Silencing PP4C in SW620 and LOVO cells led to a significant inhibition in cell proliferation (Figure 5F and G). In addition, Matrigel™ invasion assay showed that the knockdown of PP4C markedly decreased the invasiveness in SW620 and LOVO cells (Figure 5H). Three different shRNA constructs were used, and the knockdown efficiency was measured by Western blotting analysis (Figure 6).These results suggested that PP4C significantly promotes cell proliferation and invasion in CRC cells.

### PP4C medicates cell migration and invasion by increasing the expression levels of MMP-2 and MMP-9

**Figure 7:**
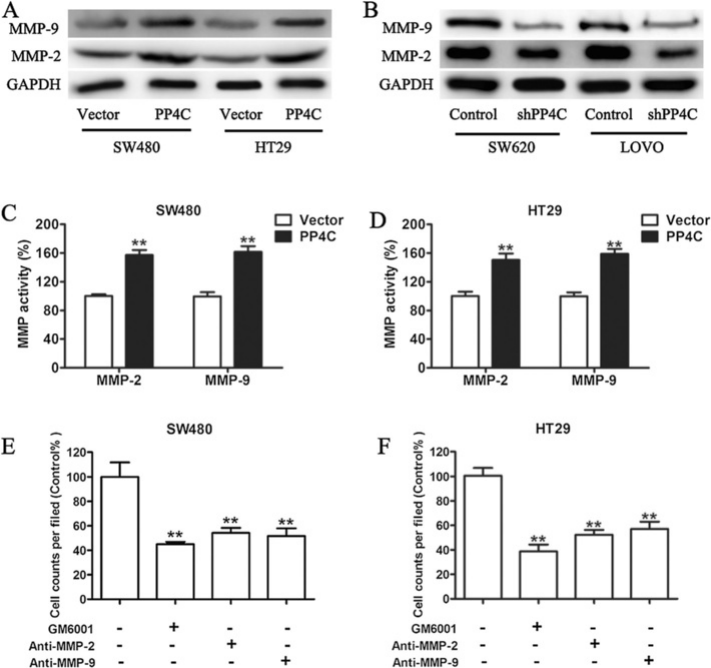
MMP-2 and MMP-9 were essential for mediating PP4C-induced cell invasion. **(A)** The protein expression levels of MMP-2 and MMP-9 were detected by immunoblotting in SW480 and HT29 cells overexpressing PP4C. **(B)** Immunoblotting of MMP-2 and MMP-9 expression in SW620 and LOVO cells expressing lentiviral PP4C shRNA. **(C, D)** Conditioned medium from SW480 and HT29 cells overexpressing PP4C was collected and evaluated using the MMP gelatinase activity assay. **(E, F)** SW480 and HT29 cells overexpressing PP4C were pretreated with the MMP inhibitor GM6001 (25), a MMP-2 neutralizing antibody (Anti-MMP2) or a MMP-9 neutralizing antibody (Anti-MMP9) for 30 min, and the cell invasion was then determined using Matrigel invasion assay. *P < 0.05 and **P < 0.01 compared with the vector controls.

To further investigate the potential molecular targets of PP4C with implication of metastasis in colorectal cancer, the expression of genes involved in tumor invasion were examined in a targeted manner. MMPs, particularly MMP-2 and MMP-9, have been frequently implicated associated with CRC invasion and are important in the invasive and metastatic processes [25-27]. The correlation between overexpressed PP4C-incuded cell invasion and the expression and activity of MMP-2 and MMP-9 were determined. Western blot indicated that the overexpression of PP4C increased MMP-2 and MMP-9 (Figure 7A). Additionally, the protein levels of MMP-2 and MMP-9 were significantly decreased when PP4C was knocked down by shRNA in SW620 and LOVO cells PP4C (Figure 7B). Consistent with our previous results, MMP gelatinase activity assay showed that the activities of MMP-2 and MMP-9 were significantly increased in PP4C-overexpressing SW480 and HT29 cells compared with the vector control group (Figure 7C and D).

To further examine the potential contribution of MMP-2 and MMP-9 in the PP4C-induced invasion of CRC cells, the gelatinase activities were blocked using the general MMP inhibitor GM6001 for both MMP-2 and MMP-9. Results indicated that the inhibition of MMP-2 and MMP-9 significantly blocked the cell invasion enhanced by PP4C overexpression (Figure 7E). Taken together, these results demonstrated that PP4C promotes cell invasion through mediating the expressions and activities of MMP-2 and MMP-9.

### PI3K/Akt signaling pathway is required for the PP4C-induced cellular invasion and activities of MMP-2 and MMP-9 in CRC cells

**Figure 8:**
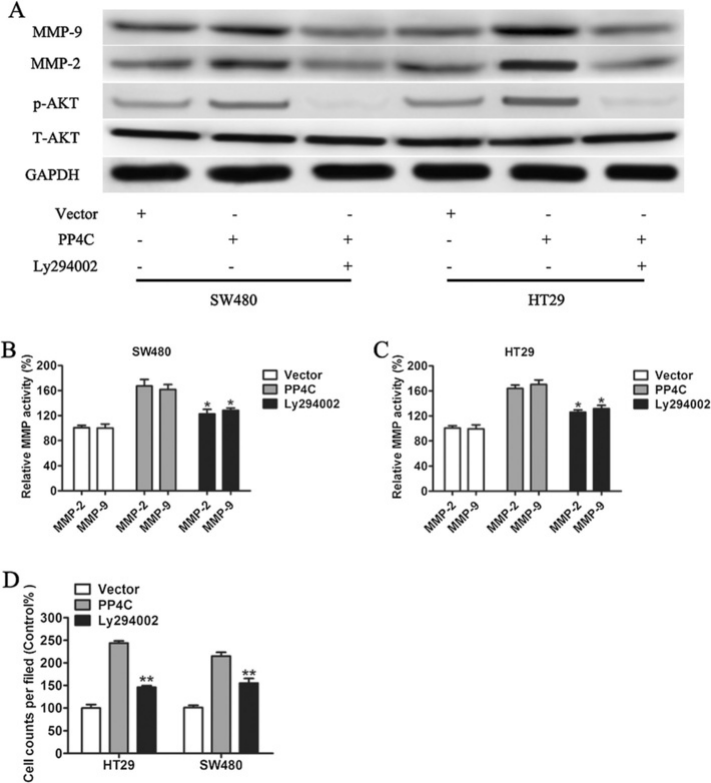
Involvement of the PI3K/AKT pathway in PP4C-induced MMP-2 and MMP-9 expression and activation and cell invasion. **(A)** Levels of p-AKT, T-AKT, MMP-2 and MMP-9 were evaluated in SW480 and HT29 cells after the indicated treatment. **(B, C)** The MMP gelatinase activity was determined after the indicated treatment. **(D)** Matrigel cell invasion was evaluated in SW480 and HT29 cells transfected with either vector control or PP4C followed by treatment with the PI3K inhibitor Ly294002 (25 μmol).

**Figure 9:**
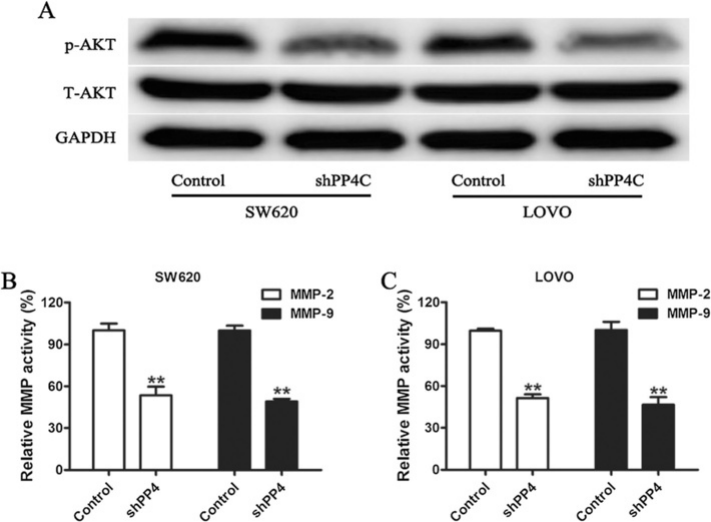
Effect of PP4C knockdown on AKT signaling molecules in CRC cells involved in MMP gelatinase activity. **(A)** Western blotting analysis of p-AKT and T-AKT expression was analyzed in PP4C-silenced SW620 and LOVO cells. **(B, C)** The MMP gelatinase activity was determined after PP4C silencing.

Because PI3K/Akt has been reported to regulate MMP-2 and MMP-9 expression, we investigated the effects of ectopic PP4C expression on the expression of p-Akt (Ser473), which is critical for the function and activity of AKT. As shown in Figure 8A, Overexpression of PP4C increased p-AKT protein expression (Figure 8A), whereas depletion of PP4C decreased p-AKT (Ser473) protein expression in both SW620 and LOVO cells (Figure 9). However, the manipulations of PP4C levels had little effect on the expression of the total protein expression level of AKT (Figures 8A and 9). As expected, PP4C silencing significantly decreased the activities of MMP-2 and MMP-9 (Figure 9).

**Figure 10:**
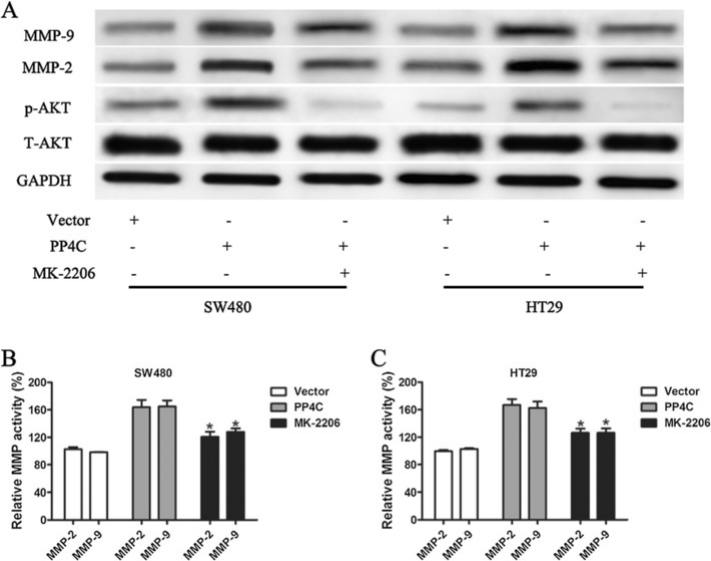
MK-2206 blocked PP4C-induced MMP-2 and MMP-9 expression and activation. **(A)** Levels of p-AKT, T-AKT, MMP-2 and MMP-9 were evaluated in SW480 and HT29 cells after the indicated treatment. **(B, C)** The MMP gelatinase activity was determined after the indicated treatment.

To further evaluate the contribution of PI3K/Akt signaling to PP4C-induced cell invasion and MMP-2 and MMP-9 expression, Ly294002, a PI3K inhibitor, was used to inhibit the activation of PI3K/AKT pathway and PP4C-induced AKT activation. Furthermore, PP4C-induced high MMP-2 and MMP-9 expression levels (Figure 8A) and activities (Figures 8B and C) in SW480 and LOVO cells were reversed by targeted inhibition using Ly294002. Consequently, Ly294002 abolished the effect of PP4C overexpression on SW480 and HT29 cell invasion (Figure 8D). Consisted with above results, depletion of PP4C decreased p-AKT (Ser473) protein expression in both SW620 and LOVO cells (Figure 9). Similarly, PP4C silencing significantly decreased the activities of MMP-2 and MMP-9 (Figure 9). We next used MK-2206, an Akt inhibitor, to block AKT activity. As shown in Figure 10, blocking Akt significantly decreased the effect on PP4C stimulation of MMP-2 and MMP-9 expression and activity. Taken together, these findings indicated that PP4C induces CRC cell invasion, production and activation of MMP-2 and MMP-9 through activation of the PI3K/AKT pathway.

### Overexpression of PP4C in SW480 cells enhances tumor growth and metastatic potential *in vivo*

**Figure 11:**
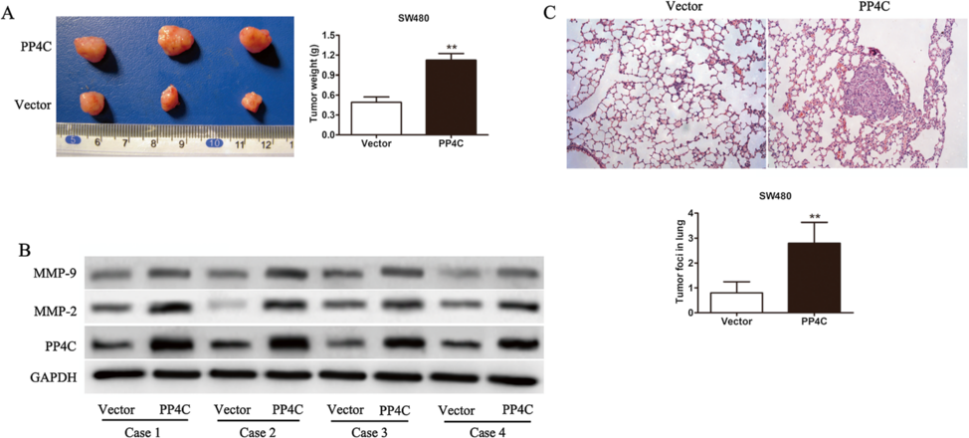
PP4C promoted CRC cell tumorigenicity and invasiveness of SW480 cells in vivo. **(A)** Representative photographs of tumors derived from SW480-PP4C or SW480-vector control cells in nude mice. The bar graphs showed the average tumor weight (gram). **(B)** At the end of the experiment, the expression of proteins of PP4C, MMP-2 and MMP-9 were determined in tissues from mice bearing SW480 xenograft tumors. **(C)** The number of tumor nodules and foci in the lungs was counted based on hematoxylin and eosin staining (Original magnification, 200 × for **B**). The values represent the means ± SD from 18 mice. **P < 0.01 (Student’s t test).

The *in vitro* experiments revealed that PP4C improves the proliferation and invasion of CRC cells. Whether PP4C can affect tumorigenicity and tumor metastasis in vivo was further investigated. The flanks of four-week-old nude mice were injected subcutaneously with SW480-PP4C cells that were stably expressing PP4C or vector-transfected cells. The sizes of growing tumors were monitored weekly for four weeks. All of the mice were sacrificed four weeks after inoculation and tumor mass was weighed. The sizes of tumor from the SW480-PP4C group were significantly larger than those from the SW480-vector group. The average tumor weights were similar (Figure 11A). To further confirm the relationship by which PP4C promote tumor growth and metastasis via upregulation of MMP-2 and MMP-9, we evaluated the expression levels of PP4C, MMP-2 and MMP-9 in vivo. As shown in Figure 11B, Western blotting showed that PP4C overexpression significantly induced MMP-2 and MMP-9 expression. Additionally, SW480-PP4C cells or vector-transfected cells were injected into the mice via the tail vein and the tumor formation in the lungs was assessed five weeks after inoculation. The number and size of the lung metastatic nodules was markedly increased in the SW480-PP4C group compared with the vector controls (Figure 11C). These results suggested that PP4C significantly promotes tumor growth and that the overexpression of PP4C markedly enhances the metastasis of SW480 cells *in vivo*, indicating that PP4C plays a positive role in CRC tumorigenicity and metastasis.

## Discussion

Previous studies have shown that PP4C is overexpressed in breast cancer, lung cancer and pancreatic ductal adenocarcinoma and has implications for tumor prognosis [16,17]. However, little is known regarding its expression pattern and exact mechanism in CRC. This study demonstrated that PP4C gene expression was increased in CRC and correlated with lymphatic nodes, distant metastasis and with overall patient survival.

To explore the possible biological functions of PP4C in CRC, we first assessed the expression of PP4C in CRC tissues and CRC cell lines at the mRNA and protein levels. Our data showed that PP4C is markedly overexpressed in CRC tissues compared with their adjacent non-tumorous tissues. The expression levels of PP4C in CRC cell lines further supported the results from the tissue samples. Interestingly, there was a significant positive correlation between PP4C expression and aggressive clinical behaviors in CRC. In addition, CRC patients with a high level of PP4C expression showed poor overall survival, as determined by a Kaplan-Meier survival assay.

It has been previously shown that PP4C is involved in mediating microtubule growth, cell migration and centrosome maturation in mitosis and meiosis [11,12]. Numerous studies have also shown that PP4C regulates the NF-κB, JNK and m-TOR pathway and is associated with cisplatin sensitivity [14,18,19]. These data demonstrated that PP4C may play an important role in cancer progression. Consistent with these results, our results suggested that PP4C acts as an oncogene in CRC. To further elaborate the biological functions in CRC, we examined the effect of PP4C on SW480 and HT29 cell proliferation and invasive activity using CCK-8 and Matrigel™ Invasion Chambers, respectively. As expected, the overexpression of PP4C promoted cell proliferation and invasion, whereas PP4C silencing inhibited cell proliferation and invasion in SW620 and LOVO cells, which present high endogenous PP4C levels. In addition, our data indicated that the overexpression of PP4C notably promotes the ability of CRC cells to form tumors and lung colonies in the *in vivo* model. These results were consistent with previous studies regarding the expression of PP4C in CRC tissues, which indicated that it may play a crucial role in phenotype behavior in a clinical study.

Cancer invasion and metastasis are multiple steps involving genetic alterations and deregulation of multiple signaling pathways [21,28,29]. The high mortality rate attributes to extensive local tumor invasion and distant metastasis [30]. Previous studies demonstrated that depletion of PP4C in HEK293 cells resulted in severely decreased cell migration and suggested that PP4C complexes may coordinate centrosome maturation and cell migration via regulation of Rho GTPases [31]. We then focused on the effects of PP4C on the invasion and metastasis in CRC cells. Increasing evidence suggests that MMPs, particularly MMP-2 and MMP-9, are upregulated in cancer cells and play a critical role in these processes [25,32,33]. In this study, we showed that upregulation of PP4C enhanced the expression and secretion of MMP-2 and MMP-9. In contrast, the knockdown of PP4C by shRNA reduced the expression and secretion of MMP-2 and MMP-9. Furthermore, the blockade of MMP-2 and/or MMP-9 reversed the stimulus effects of PP4C on cell invasion. However, further studies are needed to explain the underlining mechanisms, which contribute to the alteration of MMPs induced by PP4C. Furthermore, PI3K/AKT contributes to extracellular matrix destruction by increasing the production of MMP-2 and MMP-9 in many cancers [27,34,35]. It remains to be further investigated whether PP4C utilizes the same pathway for its effects on cell motility and invasion and also its effects on the expression and activation of MMP-2 and MMP-9. In this study, the phosphorylation of AKT was increased in PP4C-overexpressing SW480 and HT29 cells and decreased in PP4C-knockdown SW620 and LOVO cells. Extensive studies have shown that the inhibition of PI3K/AKT signaling with Ly294002 and MK-2206 abrogated cell invasion induced by PP4C and the expression and activities of MMP-2 and MMP-9. Taken together, these results suggested that the PI3K/AKT axis could be a potential oncogenic mechanism, in which PP4C contributes to the upregulation of MMP-2 and MMP-9 and cell invasion.

## Conclusions

Our results provide the first demonstration that PP4C is frequently overexpressed in CRC. A higher overexpression of PP4C is associated with the tumor phenotype and a worse outcome in CRC patients. PP4C overexpression promotes cell growth and invasion both *in vitro* and *in vivo*. The mechanisms through which PP4C acts as an oncogene in CRC involve AKT phosphorylation and upregulated MMP-2/9 expression. Although our findings of the complicated mechanisms underlying CRC progression are limited, the selective overexpression of PP4C in CRC suggests that PP4C may be an important target for therapeutic intervention.

## Methods

### Patients and samples

Total samples of 92 CRC tissues and matched adjacent non-cancer colorectal tissues were collected from patients received CRC surgery for CRC in Fudan University Cancer Hospital (Shanghai, China) from October 2003 to May 2008. Written informed consents were obtained from all of the patients. The study protocol was approved by the Clinical Research Ethics Committee of Fudan University Cancer Hospital. The diagnosis of each case was confirmed through H&E slides by two blinded expert pathologists. Immediately upon tumor excision, each tissue sample was cut into two sections: one was snap-frozen in liquid nitrogen and stored at -80°C until use (for RNA), and the other was fixed in 4% formalin and embedded in paraffin after 24 h (for immunohistochemistry). The clinicopathological features of these patients were presented and summarized in Table 1. The patient follow-up data were updated every six months by telephone. The overall survival was the time from the dates of surgery to the time of last follow-up or of death from CRC.

### Cell lines and cell culture

The human CRC cell lines (SW480, HT29, SW620 and LOVO) and the normal human intestinal epithelial cell line (HIEC) were purchased from the Culture Collection of the Chinese Academy of Sciences (Shanghai, China) and cultured in RPMI-1640 medium (Life Technologies, Shanghai, China). The HEK 293 T cell line was conserved in our laboratory and grown in Dulbecco’s modified Eagle’s medium (Life Technologies, Shanghai, China). All of the cells were grown in medium supplemented with 10% fetal bovine serum (Gibco, Carlsbad, CA, USA), 100 units/ml penicillin (Life Technologies) and 100 μg/ml streptomycin (Life Technologies) at 37°C in a humidified atmosphere containing 5% CO2. The cells were treated with GM6001 (Chemicon, Temecula, CA, USA), a broad-spectrum matrix metalloproteinase inhibitor, Ly294002 (Sigma), a selective inhibitor of PI3K, or MK-2206 (Merck, Whitehouse Station, NJ, USA), an Akt inhibitor, as indicated.

### RNA isolation and quantitative RT-PCR (qRT-PCR)

The total RNA from the tissues or cultured cells was extracted using the TRIzol reagent (Invitrogen, Carlsbad, CA, USA) according to the manufacturer’s instructions. Then, 500 ng of total RNA was reverse-transcribed in a final volume of 10 μl with PrimeScript™ II First-Strand cDNA Synthesis Kit (TaKaRa, Shiga, Japan). qRT-PCR was performed using the SYBR Green Master Mix (Roche, Mannheim, Germany) on an ABI 7900HT Fast Real-Time PCR System (Applied Biosystems, Foster City, CA, USA) in triplicate, and non-template controls were run for each assay under the same conditions. The PCR reaction was carried out with an initial denaturation step of 95°C for 10 min followed by 40 cycles of 95°C for 15 s and 60°C for 1 min. The primers for PP4C were designed using the Primer Premier 5.0™ software. The PP4C primer sequences were (forward) 5′-CTGGATCAGATTCGGACAATCG-3′ and (reverse) 5′-CACCGTCTCATTGAAGTGCCA-3′. The primer GAPDH sequences were (forward) 5′-AGCCTTCTCCATGGTGGTGAA-3′ and (reverse) 5′-ATCACCATCTTCCAGGAGCGA-3′. The results were analyzed by SDS2.3, and the relative expression was normalized to the expression of glyceraldehyde 3-phosphate dehydrogenase sequences (GAPDH). The fold change of PP4C expression was calculated as the mean relative PP4C gene expression in cancers divided by the mean of relative KIF2A gene expression in adjacent normal tissues.

### Immunohistochemical staining

Formalin-fixed and paraffin-embedded sections with a thickness of 4 μm were deparaffinized, rehydrated and washed in PBS. The sections were then incubated in 0.01 M sodium citrate buffer (pH 6.0) for 30 min, 3% H2O2 for 30 min (to block endogenous peroxidase activity), and 10% normal goat serum for 30 min (to block nonspecific staining). The primary antibody, namely anti-PP4C rabbit polyclonal antibody (OriGene, Rockville, MD, USA), was applied in a humidified chamber at 4°C overnight. The sections were then incubated with the secondary antibody (anti-rabbit antibody) and then with streptavidin-POD (DAKO, Glostrup, Denmark). The specific antibody binding was visualized using DAB solution (DAKO). The tissues were counterstained with hemalaun solution (DAKO). The primary antibody was replaced by PBS, which was used as a negative control.

The degree of immunostaining of the sections was viewed and scored separately by two pathologists who were blinded to the clinical and histopathologic features. The scores were assigned to combine the proportion of positively stained tumor cells and the staining intensity. The number of positive tumor cells was graded as follows: ‘-’ (< 5% positive tumor cells), ‘+’ (5-25% positive tumor cells), ‘++’ (26-50% positive tumor cells), and ‘+++’ (> 50% positive tumor cells). The staining intensity was recorded as follows: ‘-’ (no staining), ‘+’ (weak staining, light yellow), ‘++’ (moderate staining, yellowish brown), and ‘+++’ (strong staining, brown). For survival data analysis, the samples scored ‘–’ or ‘+’ were considered to present a low level of PP4C expression, and the samples scored ‘++’ or ‘+++’ were considered to present a high level of PP4C expression.

### Construction of vectors

The full-length CDS of human PP4C was amplified from the cDNA from HEK293T cells using the following forward and reverse primers: 5′-AAATCTAGAATGGCGGAGATCAGCGAC-3′ and 5′-AGCGGCCGCCTTCCCAGCGAAATCATC -3′, respectively. The PCR product was purified using a PCR purification kit (Qiagen Chatsworth, CA, USA) and then cloned into the XhaI and BamHI sites of the pCDH-CMV-MCS-EF1-Puro vector (System Biosciences, Mountain View, CA, USA). Successful cloning was confirmed by sequencing. To knock down PP4C expression, short hairpin RNA (shRNA) sequences specific to PP4C (clone 1-TRCN0000272744, clone 2-TRCN0000272745, and clone 3-TRCN0000272746) were purchased from Sigma-Aldrich (Sigma-Aldrich, St. Louis, MO, USA).

### Lentiviral infections

The lentivirus-mediated PP4C packaging vectors or pCDH-CMV-MCS-EF1-Puro vectors were co-transfected with the packaging vectors into HEK293T cells using the Fugene HD Reagent (Roche, Basel, Switzerland) to produce virus following the manufacturer′s protocol. The PP4C shRNA insert (shPP4C) or a scrambled sequence (Control) were co-transfected with Δ8.9 and Vsvg at a ratio of 4:3:2 in quantity into HEK293T cells using the Fugene HD Reagent (Roche). Forty-eight hours following transfection, the infection-competent viral supernatants were collected and used to infect cells with 8 μg/ml polybrene (Sigma-Aldrich). The stable clones were then selected in a medium containing puromycin (5 μg/ml; InvivoGen, San Diego, CA, USA). Seventy-two hours after infection, the cells were selected to determine the expression of PP4C before the experiments.

### Cell Count Kit-8 (CCK-8) assay and invasion assays

For the cell proliferation assays, we used the Cell Counting Kit-8 assay (CCK-8; DOJINDO, Tokyo, Japan) according to the manufacturer’s instructions. The cells were seeded into 96-well plates at 4 × 103/well. At the indicated time, 10 μl of CCK-8 was added to each well, which contained 100 μl of medium. After incubation for 2 h, the optical densities at 450 nm of each well were measured using a microplate reader (Model 680 Microplate Reader; Bio-Rad, Richmond, CA, USA). Each sample had four duplicate wells and was independently performed in triplicate.

The cell invasion was assessed using 24-well poly-carbonate filters (membrane pore size 8 μm; Costar, Cambridge, MA, USA). The upper surface of each insert was coated with 100 μl per well of diluted matrigel (BD Biosciences, Bedford, MA, USA) (1:5), and the bottom wells were filled with complete medium (10% fetal bovine serum) as chemoattractant following the manufacturer’s protocol. Then, 5 × 104 cells were suspended in 100 μl of serum-free medium and seeded into the upper chamber of the 24-well transwell inserts. After 24 h of incubation at 37°C, the non-invading cells were mechanically removed from the upper surface of the membrane by wiping with cotton swabs. The cells on the lower surface of the membrane were fixed with methanol and stained with 1% crystal violet for 15 min. The stained cells were counted in five randomly fields per filter under a microscope (100× magnification). The experiments were performed in triplicate wells and independently repeated three times.

### MMP gelatinase activity assay

Each group of cells was incubated in serum-free medium with or without treatment for 24 h. The activities of MMP-2 and MMP-9 in the conditioned culture supernatant were determined with the CHEMICON Gelatinase Activity Assay Kit (Chemicon, Temecula, CA, USA) according to the manufacturer’s protocols.

### *In vivo* tumor growth and metastasis study

All of the procedures involving animals were performed according to the NIH Guide for the Care and Use of Laboratory Animals and local institutional ethical guidelines for animal experimentation, and the protocols were approved by the Experimental Animal Ethics Committee of Fudan University Shanghai Medical College with permit number 20130148 F. Four-week-old female BALB/c athymic nude mice were purchased from Slaccas (Slaccas Laboratory Animal, Shanghai, China). SW480 cells (5 × 106 cells/mouse) infected with vectors or PP4C in 150 μl of FBS-free medium were injected subcutaneously into the flank region of the mice. Tumor measurements were taken with calipers once weekly, and the tumor volume (V) was calculated using the following formula: (width2 × length)/2. For lung metastasis formation, we used six-week-old nude mice for the tail vein metastasis model. SW480 cells (5 × 105 cells/mouse) infected with vectors or PP4C in 100 μl of sterile PBS were separately injected into the tail vein of each mouse. The animals were maintained in a sterile animal facility and killed six weeks later. The tumors were excised, weighed and measured. The presence of metastatic lesions in the lungs was determined using a dissecting microscope.

### Western blotting

The cells were lysed with RIPA lysis buffer from Cell Signaling Technology (CST, Beverly, MA, USA). Equal amounts of protein were separated using 10% SDS-PAGE and transferred to a PVDF membrane (Millipore, Bedford, MA, USA). The membranes were blocked with 5% nonfat dry milk in TBST buffer at room temperature for 1 h and then incubated overnight at 4°C with the primary antibodies against PP4C (OriGene), phospho-AKT (Ser473), AKT, MMP-2 and MMP-9 (from CST). An antibody to GAPDH from Kangchen (Kangchen, Shanghai, China) was used as a loading control. After washing, the membranes were incubated with HRP-conjugated secondary antibodies (Kangchen) for 1 h at room temperature and finally visualized using the SuperSignal® West Pico chemiluminescent substrate kit (Pierce, Rockford, IL, USA). The images were captured using a luminescent image analyzer LAS-4000 mini (FujiFilm, Tokyo, Japan).

### Statistical analysis

The significance of the data from patient specimens was determined by the χ2 test, and the overall survival rates were determined using the Kaplan-Meier method. The significance of the *in vitro* data and *in vivo* data was determined by a two-tailed independent sample student’s t-test. ANOVA was employed to compare the differences among multiple cell lines treated as indicated. The statistical analyses were performed using the GraphPad Prism 5 software package (GraphPad software Inc, San Diego, CA, USA), and P < 0.05 was considered to be significant.

## Abbreviations

CCK-8: Cell count kit-8
DMEM: Dulbecco’s modified Eagle’s medium
FBS: Fetal calf serum
IHC: Immunohistochemistry
OD: Optical density
CRC: Colorectal carcinoma
qRT-PCR: Quantitative reverse transcription polymerase chain reaction
RT: Reverse transcription
